# Dentatorubrothalamic tract localization with postmortem MR diffusion tractography compared to histological 3D reconstruction

**DOI:** 10.1007/s00429-015-1115-7

**Published:** 2015-10-05

**Authors:** J. Mollink, K. M. van Baarsen, P. J. W. C. Dederen, S. Foxley, K. L. Miller, S. Jbabdi, C. H. Slump, J. A. Grotenhuis, M. Kleinnijenhuis, A. M. van Cappellen van Walsum

**Affiliations:** 1 Nuffield Department of Clinical Neurosciences, FMRIB Centre, University of Oxford, Oxford, UK; 2 Department of Neurosurgery, Radboud University Medical Centre, Nijmegen, The Netherlands; 3 Department of Anatomy, Donders Institute for Brain Cognition and Behaviour, Radboud University Medical Centre, Nijmegen, The Netherlands; 4 MIRA Institute for Biomedical and Technical Medicine, University of Twente, Enschede, The Netherlands

**Keywords:** Diffusion-weighted imaging, Tractography, Postmortem, Histological reconstruction, Cerebellum, Dentatorubrothalamic tract

## Abstract

Diffusion-weighted imaging (DWI) tractography is a technique with great potential to characterize the in vivo anatomical position and integrity of white matter tracts. Tractography, however, remains an estimation of white matter tracts, and false-positive and false-negative rates are not available. The goal of the present study was to compare postmortem tractography of the dentatorubrothalamic tract (DRTT) by its 3D histological reconstruction, to estimate the reliability of the tractography algorithm in this specific tract. Recent studies have shown that the cerebellum is involved in cognitive, language and emotional functions besides its role in motor control. However, the exact working mechanism of the cerebellum is still to be elucidated. As the DRTT is the main output tract it is of special interest for the neuroscience and clinical community. A postmortem human brain specimen was scanned on a 7T MRI scanner using a diffusion-weighted steady-state free precession sequence. Tractography was performed with PROBTRACKX. The specimen was subsequently serially sectioned and stained for myelin using a modified Heidenhain–Woelke staining. Image registration permitted the 3D reconstruction of the histological sections and comparison with MRI. The spatial concordance between the two modalities was evaluated using ROC analysis and a similarity index (SI). ROC curves showed a high sensitivity and specificity in general. Highest measures were observed in the superior cerebellar peduncle with an SI of 0.72. Less overlap was found in the decussation of the DRTT at the level of the mesencephalon. The study demonstrates high spatial accuracy of postmortem probabilistic tractography of the DRTT when compared to a 3D histological reconstruction. This gives hopeful prospect for studying structure–function correlations in patients with cerebellar disorders using tractography of the DRTT.

**Electronic supplementary material:**

The online version of this article (doi:10.1007/s00429-015-1115-7) contains supplementary material, which is available to authorized users.

## Introduction

Tractography, based on diffusion-weighted imaging (DWI), is a technique with great potential to characterize the in vivo anatomical position and integrity of white matter tracts (Basser et al. 2000; Behrens et al. 2003; Mori et al. 1999). Tractography has proven its worth in neuroscience as well as in neurology and neurosurgery (Bick et al. 2012; Dimou et al. 2013; Potgieser et al. 2014). It is an invaluable tool in investigating structure–function relationships.

The white matter tract of our interest is the dentatorubrothalamic tract (DRTT). This tract originates in the dentate nucleus of the cerebellum and projects to the ventrolateral nucleus of the thalamus via the red nucleus. Left and right tracts decussate in the mesencephalon at the level of the inferior colliculi. Neuroscience of the cerebellum is gaining attraction and as the DRTT is the main output tract of the cerebellum, it is currently subject to many clinical and neuroimaging studies.

During the last two decades, evidence for a role of the cerebellum in cognition, language and emotional processing is growing (De Smet et al. 2013; Mariën et al. 2014; Stoodley and Schmahmann 2009; Stoodley 2012; Strick et al. 2009). Anatomical studies have shown that cerebellar cortical areas are reciprocally connected with premotor, parietal and temporal cerebral cortex (Dum and Strick 2003; Strick et al. 2009).

In mammals, the cerebellar cortex, the cerebellar nuclei and the white matter in between seem to be segregated into distinct functional zones (Nieuwenhuys et al. 2008; Ramnani 2006). Additionally, functional MRI studies have shown that there is a topographical organization in the cerebellar cortex, not only regarding motor control but also regarding cognitive and affective functions (Stoodley and Schmahmann 2009, 2010). Furthermore, there is clinical evidence that supports a cerebellar role in non-motor function. Patients with cerebellar lesions (bleeding, infarction or post-surgery) may suffer from cognitive, language and emotional disturbances (De Smet et al. 2011; Küper and Timmann 2013; Schmahmann and Sherman 1998; van Baarsen and Grotenhuis 2014). Likewise, psychiatric disorders such as autism are found to be correlated with cerebellar structural anomalies (Becker and Stoodley 2013).

A striking example of cerebellar dysfunction is the cerebellar mutism syndrome. This rare but devastating syndrome may occur in children after surgery for a cerebellar tumour and causes an inability to speak in addition to emotional lability and behavioural disturbances (Küper and Timmann 2013; Reed-Berendt et al. 2014). The syndrome may occur even in a telovelar approach when there seems to be no damage to the cerebellar cortex or white matter.

The exact pathophysiology and the anatomical substrate of non-motor disorders caused by cerebellar dysfunction are still not fully understood. The cerebellum is, therefore, subject to many clinical and neuroimaging studies. It seems that the DRTT plays an important role as it is the main cerebellar output tract. Better understanding of structure–functional relationships in the cerebellum and its output tracts may lead to a better understanding of cerebellar pathology and in the end, better treatment or even prevention.

Tractography offers the in vivo investigation of the anatomical position of white matter tracts, their volume and integrity. However, although the technique is valuable, its anatomical accuracy is still poorly determined while its limitations are well known. Before using tractography as a tool in investigating the integrity of the DRTT, its correspondence to known anatomy should be investigated. In the cerebellum, the DRTT is enveloped by white matter of the inferior and middle cerebellar peduncles. At the level of the brainstem, it is surrounded by the brachium of the inferior and superior colliculus, medial longitudinal fasciculus and the central tegmental tract (Naidich et al. 2009). It is the question whether tractography is able to correctly distinguish between these tracts.

The precision and reliability of tractography results are largely influenced by image quality, parameter settings and even choice of tracking algorithms (Barbieri et al. 2011; Feigl et al. 2014). Together with the low SNR, this implies that many false positives and false negatives might occur in the tracking process (Jbabdi and Johansen-Berg 2011). In addition, compared to the cerebral hemispheres, tractography of cerebellar white matter pathways faces particular challenges. Air–tissue interfaces are in closer proximity to the structures of interest, white matter tracts are smaller, tract curvatures are stronger and there are many kissing and crossing fibres in the cerebellum (Habas and Cabanis 2006, 2007; Kwon et al. 2011).

To date, the general accuracy of tractography remains undetermined and false-positive and false-negative rates are not available (Chung et al. 2011). Various methods were applied previously to study the anatomical reliability of tractography. These include comparison to neuroanatomical tracing, fibre dissection and histology. This kind of comparison was reported by a handful of studies, but is beginning to gain traction as tractography becomes more widely used.

A few studies (Fernandez-Miranda et al. 2012; Holl et al. 2011; Lawes et al. 2008) considered classical dissection of white matter fibres as the anatomical reference. In general, a close correspondence between reconstructed tracts and anatomical dissection was reported in these studies. However, tractography was based on in vivo DWI data and, therefore, lacking a direct and quantitative comparison with anatomical dissection in the specimens.

Neuroanatomical tracing provides another way of mapping neural networks by injecting tracing dyes that diffuse along axonal trajectories. In macaque monkeys it was shown that there was generally good agreement between the anatomical position of tracts in the corticospinal tract as assessed with tractography when compared to neural tracers (Dauguet et al. 2007). Similar results were shown in mini pigs (Dyrby et al. 2007). To bridge the gap between animals and humans, ventral prefrontal cortex tracts were inferred with tractography in both humans and macaques (Jbabdi et al. 2013). Tractography in macaques was validated with neural tracing before a comparison was made between primate and human tractography. A high spatial similarity was found between the two techniques. Furthermore, white matter trajectories in macaques were manifested in a similar manner as those in the human brain. The problem with neural tracers, however, is that they are largely limited to animal studies. Even though some tracers are applicable in postmortem human brain tissue, they require infiltration times that can take up to several years (Seehaus et al. 2013). In addition, neural tracers are very useful for mapping individual axons and their projections that intermingle with larger bundles in the central nervous system (CNS). Tractography, however, lacks such detailed axonal discrimination and generally determines tract borders. To obtain false-positive and false-negative rates, neural tracing may not be the most appropriate method to determine the spatial accuracy of tractography.

The present study considered histological sectioning and myelin staining as the gold standard for mapping white matter tracts. In general, histology offers a resolution that is far beyond that of DWI and enables a more precise delineation of fibre tracts (Bürgel et al. 2006). Traditional myelin stains often do not provide contrast to distinguish between densely packed white matter bundles. Here, a modified Heidenhain–Woelke staining protocol was applied to visualize myelinated white matter. The modification of this protocol inactivates the chromatogen complexes in the thinnest myelin sheaths. This produces a graded reduction in myelin staining in white matter that appears to be proportional to the amount myelination (Bürgel et al. 1997, 1999, 2006).

The main goal of the present study was to investigate the correspondence between tractography of the dentatorubrothalamic tract with its anatomy as known from a three-dimensional histological reconstruction of this tract visualized with the modified Heidenhain–Woelke stain in the same postmortem specimen.

## Methods

### Sample acquisition

For this study, the brain of an 87-year-old woman was acquired via the body donor program at the Department of Anatomy of the Radboud University Medical Centre, Nijmegen, The Netherlands. The subject died from pneumonia and had no prior neurological or psychiatric diseases. As determined by two neuroanatomists, gross morphology of the brain, as well as the serial sections, showed no signs of pathology. After 10 h of postmortem, the body was perfused via the femoral artery to allow rapid fixation of the tissue. Approximately 24 h later, the brain was extracted from the skull and stored in 7.7 % formalin for 16 months. Frontal, temporal, parietal and occipital lobes were removed to fit the specimen in a smaller MR coil for high signal reception (Supplementary Materials, Figure 8).

### Magnetic resonance image acquisition

Prior to scanning, the specimen was soaked in phosphate-buffered saline for 72 h to remove the formalin from the tissue, as formalin is known to decrease the T_2_ relaxation rate of tissue (Shepherd et al. 2009). In addition, compared to in vivo experiments fixed tissues suffer from reduced apparent diffusion coefficients (ADC) (D’Arceuil et al. 2007; Sun et al. 2003, 2005), requiring higher *b* values to obtain similar diffusion contrast as for in vivo. Recent studies also reported a subtle reduction for the fractional anisotropy (FA) in white matter of fixed brains (D’Arceuil and de Crespigny 2007; Schmierer et al. 2008). Here, a relatively short postmortem interval was employed to limit the reduction in ADC and FA (D’Arceuil and de Crespigny 2007). Further, diffusivity measures were suggested to remain stable up to a 3-year period after fixation (Dyrby et al. 2011).

All imaging was performed in a single overnight session on a Siemens MAGNETOM 7T MRI scanner (Siemens, Erlangen, Germany) with a 28-channel knee coil. Background signal was avoided by placing the specimen in tight-fitting plastic bags containing Fomblin (Solvay Solexis Inc.), a hydrogen-free liquid closely matching the susceptibility of brain tissue.

Diffusion-weighted images were acquired with a DW-SSFP (diffusion-weighted steady-state free precession) sequence (McNab et al. 2009) at 1 mm isotropic resolution with an effective *b* value of 5175 s/mm^2^ in 49 directions (2 averages). The DW-SSFP sequence has been demonstrated to provide improved tractography in postmortem brains in comparison to the more conventional diffusion-weighted spin echo due to its ability to achieve strong diffusion weighting without unacceptable T_2_ signal loss (Miller et al. 2012). Because T_1_ and T_2_ estimates are required for the analysis of the DW-SSFP data, true inversion recovery (TIR) and turbo spin echo (TSE) were included in the protocol. These techniques have recently been adapted for use at 7T using a single-line (rather than segmented EPI) 3D readout (Foxley et al. 2014), resulting in improved SNR with robust estimation of multiple fibre populations within a given voxel. An additional high-resolution structural scan with mixed contribution T_1_ and T_2_ weighting was acquired with a TRUFI (true fast imaging with steady-state free precession) sequence (Miller et al. 2011; Zur et al. 2005). Parameters are presented in Table 1.

**Table 1:** MRI scan parameters

Diffusion-weighted steady-state free precession (DW-SSFP)	
*T*_E_	21 ms
*T*_R_	30 ms
*α*	30°
Number of directions	49
Number of averages	2
Number of *q* = 10 mm^−1^ (*b* = 0 equivalent)	8
Matrix size	176 × 120 × 180 mm^3^
Voxel size	1.0 × 1.0 × 1.0 mm^3^
Bandwidth	80 Hz/pixel
*q* value	300 mm^−1^
*b* value (equivalent)	~5175 s/mm^2^
True inversion recovery (TIR, T1 quantification)	
*T*_E_	12 ms
*T*_R_	1000 ms
*T*_I_	Varying: 31, 62, 125, 250, 500 and 850 ms
Matrix size	176 × 120 × 192 mm
Voxel size	1.0 × 1.0 × 1.4 mm
Bandwidth	200 Hz/pixel
Turbo spin echo (TSE, T2 quantification)	
*T*_E_	Varying: 14, 28, 42, 55, 69, 83 and 111 ms
*T*_R_	1000 ms
Matrix size	176 × 120 × 192 mm
Voxel size	1.0 × 1.0 × 1.4 mm
Bandwidth	130 Hz/pixel
True fast imaging with steady-state free precession (TRUFI, anatomical)	
*T*_E_	3.79 ms
*T*_R_	7.58 ms
*α*	35°
Matrix size	416 × 256 × 512 mm
Voxel size	0.4 × 0.4 × 0.5 mm
Bandwidth	296 Hz/pixel

### Probabilistic tractography

Probability distributions for fibre orientations in each voxel were estimated with BEDPOSTX (Behrens et al. 2007), modified to incorporate the DW-SSFP signal equations (McNab and Miller 2008; McNab et al. 2009). More precise, the modified BEDPOSTX version includes T_1_, T_2_, and B_1_ information to allow for accurate voxel-wise estimates of diffusion coefficients. Three diffusion directions per voxel were modelled, with online model selection (automatic relevance determination, ARD) on the second and third fibre (Behrens et al. 2007). Registration between the structural space and diffusion space was performed using a 12-degree of freedom (DOF) affine transformation determined by FLIRT (Jenkinson and Smith 2001). Seed masks were manually drawn in the structural MRI for both dentate nuclei using ITK-SNAP (Yushkevich et al. 2006). White matter surrounded by the dentate nuclei was included in the segmentation. Both thalami were manually segmented and defined as target masks. The thalami were easily distinguished from the internal capsule by contrast differences between white and grey matter in the TRUFI structural MRI. Medial and lateral geniculate nuclei were included in the segmentation. An exclusion mask midsagittal through the cerebellar vermis and mid-pons was defined to prevent streamlines from crossing the midline below the level of the decussation (Supplementary Materials, Figure 9). Tractography was performed with PROBTRACKX (Behrens et al. 2007). Streamlines were generated for each voxel in the dentate nucleus seed mask that also passed the contralateral thalamic target mask (i.e. also the termination point), producing a connectivity map for each DRTT. Additional parameters included a step length of 0.5 mm, 2000 streamlines per seed voxel, a 0.2 curvature threshold (equivalent to ~78°) and no FA threshold.

Each voxel in the connectivity maps indicated the number of streamlines that passed through that particular voxel. Connectivity maps were normalized by dividing each voxel with the total number of streamlines that was generated between the seed mask and target mask.

### Tissue processing

Comparison of histology and tractography was only performed for the DRTT in the cerebellum and brainstem. This part was separated from the supratentorial part of the specimen with a transverse cut inferior to the red nuclei. The specimen, including the cerebellum and the brainstem, was embedded in paraffin before it was sectioned with an LKB 2260 Macrotome (LKB Instruments, Bromma, Sweden). The knife was positioned at a 15° angle with respect to the cutting plane. The tissue was serially sectioned at 10 µm thickness and every 20th slice was kept for staining, resulting in an inter-plane resolution of 200 µm. A total number of 202 sections were collected. Prior to cutting each third last section in a series, a block face image [i.e. a photograph of the cutting surface of the block (Amunts et al. 2013; Annese et al. 2006; Dauguet 2011; Toga et al. 1994)] was taken with a Canon EOS 550D camera with a Canon 100 mm autofocus lens. Successfully collected sections were matched with the corresponding block face. Each section was subsequently mounted on a glass plate, dried overnight in a stove at 37 °C. For optimal visualization and differentiation between degrees of myelination within white matter fibre bundles, the modified Heidenhain–Woelke stain (Bürgel et al. 1997; Holl et al. 2011) was used. Macrophotographs of the stained sections were taken with the same camera to produce digitized data. These are referred to as “histological slices” hereafter.

### Histological 3D reconstruction

Custom software was written for pre-processing, registration and 3D reconstruction of histological slices in Matlab 2013a (The MathWorks Inc, Natick, MA, USA). Prior to registration, histological slices were down sampled to match a square pixel size of 30 μm/pix. After converting the images to grayscales, contrast was enhanced by stretching the histogram and images were segmented based on edge segmentation and manual adjustment. Slice-by-slice 2D landmark-based affine registration was performed. At least six corresponding landmarks in the histological slice and its accompanying block face were selected to compute the affine transformation. Block faces serve as an intermodality that aims to preserve curvature of a volume. The so-called banana problem (Malandain et al. 2004) is introduced if curved objects are reconstructed based on inter-slice alignment and may end up as straightened objects. The presence of a reference volume, here both a block face volume and a structural MRI, prevents such reconstruction bias and retrieves the original curvature. Following affine registration to the block faces, stacking of the histological slices resulted in an initial histological 3D reconstruction.

The structural MRI was transformed to the initial histological volume with a 12 DOF, 3D affine registration implemented in FLIRT (Jenkinson and Smith 2001), using normalized mutual information as a cost function. At this stage, histological slices were matched with corresponding MRI slices. Prior to non-linear registration, a 2D 6 DOF affine transform (FLIRT) was applied to refine each histological slice with the corresponding MRI slice.

Following affine co-registration a non-linear registration approach was chosen to accurately align histological slices. Steps in tissue processing such as paraffin embedding and microtome cutting inevitably cause intrinsic deformations to each tissue section. The affine transformation model is by definition unable to correct for such tissue deformations as it assumes a single global deformation per slice. The deformations present in the histological slices, however, are often spatially variable and may differ between types of tissue (e.g. between grey and white matter). The advanced normalization tools (ANTS) (Avants et al. 2011) were applied to perform non-linear registration. A multivariate approach was chosen to align the slices in the histological volume as previously described by (Adler et al. 2014) for 3D reconstruction of hippocampal sections. In brief, for each histological slice a warp field was computed based on the two neighbouring histological slices and the corresponding MRI slice. Mutual information was used as a cost function for both the neighbouring histological slices and the MRI slice. The warps were computed using a symmetric normalized diffeomorphic transformation model (Avants et al. 2008). Histological slices were warped to their new space according to the computed warp fields at each step. The total non-linear registration approach was iterated until there was no visual difference present between consecutive steps, which was satisfactory after 20 iterations. The histological volume had a voxel size of 0.03 × 0.03 × 0.20 mm. At last, the MRI was non-linearly transformed to histological space for final refinement between the two modalities. Again, the transformation was computed using a symmetric normalized diffeomorphic transformation model.

Tract segmentation was achieved by manually drawing a polygon around the DRTT in each histological slice (Yushkevich et al. 2006). Two investigators with a good anatomical knowledge of the DRTT executed the segmentation (J. M. & K.vB.).

### Spatial tract analysis

Probabilistic tractography resulted in connectivity maps for the DRTT between the dentate nucleus and the thalamus. The concordance between the binary tractography connectivity maps (after thresholding) and the reference (i.e. the DRTT segmentation in the histological space) was computed. It was evaluated using ROC (Receiver Operating Characteristic) analysis. In the present context, an ROC curve illustrates the overlap between the tractography and histological DRTT masks. This is achieved by varying the segmented volume in the tractography map through thresholding at different connectivity values. These values range from 0 to 0.35 % of the total number of streamlines generated between the seed and target mask. For each binary connectivity map, true-positive (TP; tractography-positive and histology-positive), true-negative (TN; tractography-negative and histology-negative), false-positive (FP; tractography-positive but histology-negative) and false-negative (FN; tractography-negative but histology-positive) voxels were computed. True-positive rates (TPR), equal to sensitivity and false-positive rates (FPR), equal to 1-specificity were calculated as

1 $${\text{TPR}} = \frac{\text{TP}}{{{\text{TP}} + {\text{FN}} }}$$

2 $${\text{FPR}} = \frac{\text{FP}}{{{\text{FP}} + {\text{TN}}}}$$

TPR is represented as a function of FPR in an ROC curve.

Correspondence between the binary masks was also evaluated with the Dice similarity index (SI) (Dice 1945). The SI is a measure for correctly classified tractography voxels relative to the total volume occupied by the tractography mask plus the reference mask. It thus indicates the ratio of true-positive voxels over all voxels included by both modalities, ranging from 0 to 1.

3 $${\text{SI}} = \frac{{2 \times ({\text{Trac}} \cap {\text{Ref}})}}{{{\text{Trac }} + {\text{Ref}}}}$$

Here, Trac denotes the volume of the binary tractography mask thresholded at different connectivity values (equal to those used in ROC analysis) and Ref is the volume of the segmentation of the tract in the histological volume. The ∩ operator indicates overlapping volume of Trac and Ref.

An additional analysis was done in which the amount of false-negative voxels (i.e. voxels that were “missed” by tractography) was calculated for expanding tractography masks, to define the spatial extent of the outliers. For each mask, the number of FNs (voxels missed by tractography) was computed as the mask increased in size (in steps of one voxel at a time) by means of binary dilation.

Analyses were performed for the total tract and for three separate regions of interest (ROIs): the dentate nucleus, the superior cerebellar peduncle and the decussation of the DRTTs in the mesencephalon. The outcomes were computed for each investigator separately, after which they were averaged.

## Results

**Fig. 1:**
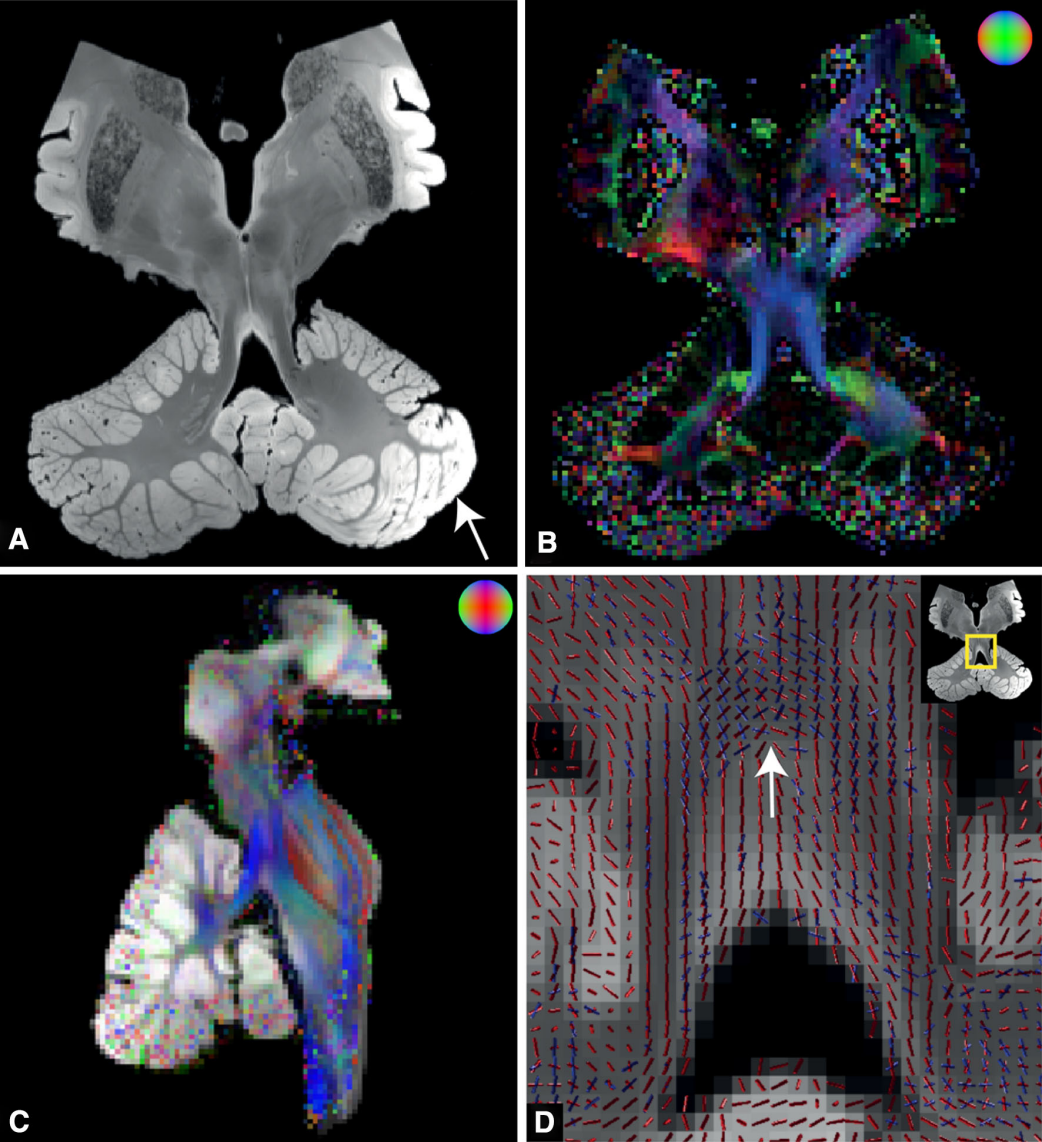
Postmortem MRI acquisitions. **a** Coronal view of the structural image acquired with the TRUFI sequence. *White arrow* inhomogeneous signal intensity in the cerebellar cortex. **b** Direction encoded colour (DEC) map with fractional anisotropy modulated intensity. Colour coding: *green* anterior–posterior, *red* left–right, *blue* inferior–superior. **c** The registration accuracy between structural and diffusion space is illustrated by overlaying DEC map with the structural MRI (sagittal view). The structural MRI was transformed to diffusion space with a 3D affine transformation. **d** ROI of diffusion directions in the decussation of the DRTTs (*white arrow*). *Red* and *blue lines* the first and second diffusion direction within a voxel, respectively

Postmortem MR images of the specimen were acquired with a total scanning time of approximately 32 h. High contrast was present between white and grey matter. Basal ganglia and cerebellar nuclei were clearly visible, and in particular the dentate nucleus showed excellent contrast with adjacent white matter (Fig. 1a). Although neither macroscopic inspection of the specimen, nor its sections had shown any sign of pathology, an inhomogeneous intensity pattern was observed throughout the cerebellar cortex in the structural MRI. The cortical artefact was not present in white matter and diffusion parameters in the white matter areas were coherent with the expected underlying anatomy.

Diffusion parameters in the white matter were successfully estimated by a modified version of the BEDPOSTX software written for the DW-SSFP sequence (McNab and Miller 2008; McNab et al. 2009). Although three diffusion directions were modelled inside a voxel, in practice, the third diffusion direction was negligible. The second diffusion direction, however, contributed considerably to model the underlying white matter architecture according to the ARD criterion (Behrens et al. 2007). This is particularly evident in regions with crossing fibres such as the decussation of the DRTTs (Fig. 1d).

**Fig. 2:**
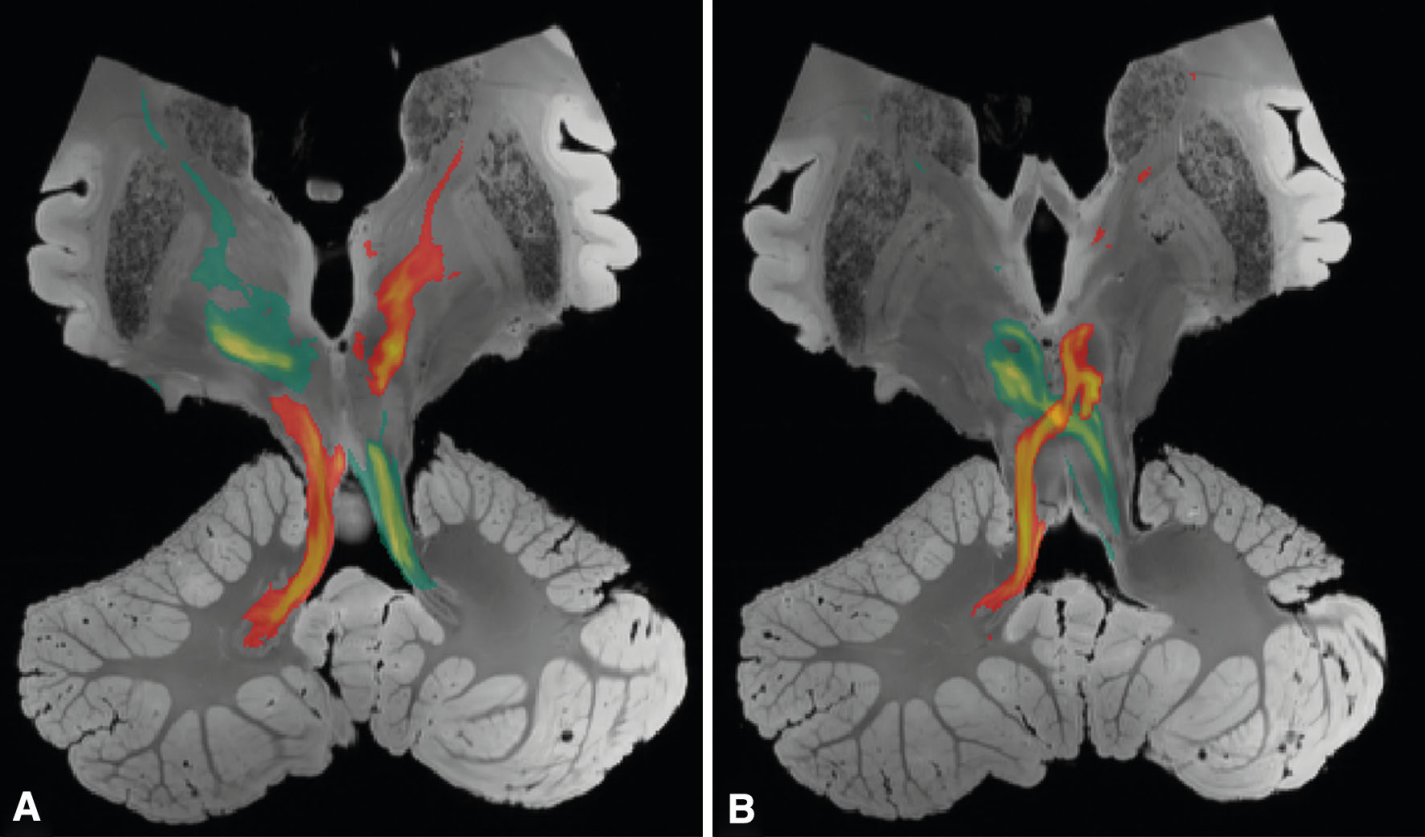
Probabilistic tractography of the DRTTs overlaid on the structural image (coronal view). *Green* and *red* tracts originate from the *left* and *right* dentate nucleus, respectively. The slice in (**a**) is located 3.1 mm anterior to the slice (**b**)

The tractography algorithm yielded well-defined most likely pathways between the dentate nuclei in the cerebellum and the contralateral thalami (Fig. 2). The tracts displayed a high degree of symmetry. From the seed region in the dentate nucleus, the reconstructed tract exited the hilum of the dentate nucleus to enter the superior cerebellar peduncle. The crossing to the contralateral side occurs at the level of the mesencephalon for both the left and right DRTT. Superior from the decussation, the DRTT traverses and encapsulates the red nuclei. Then, the tracts enter the internal capsule and branch out into their target regions in the thalamus.

**Fig. 3:**
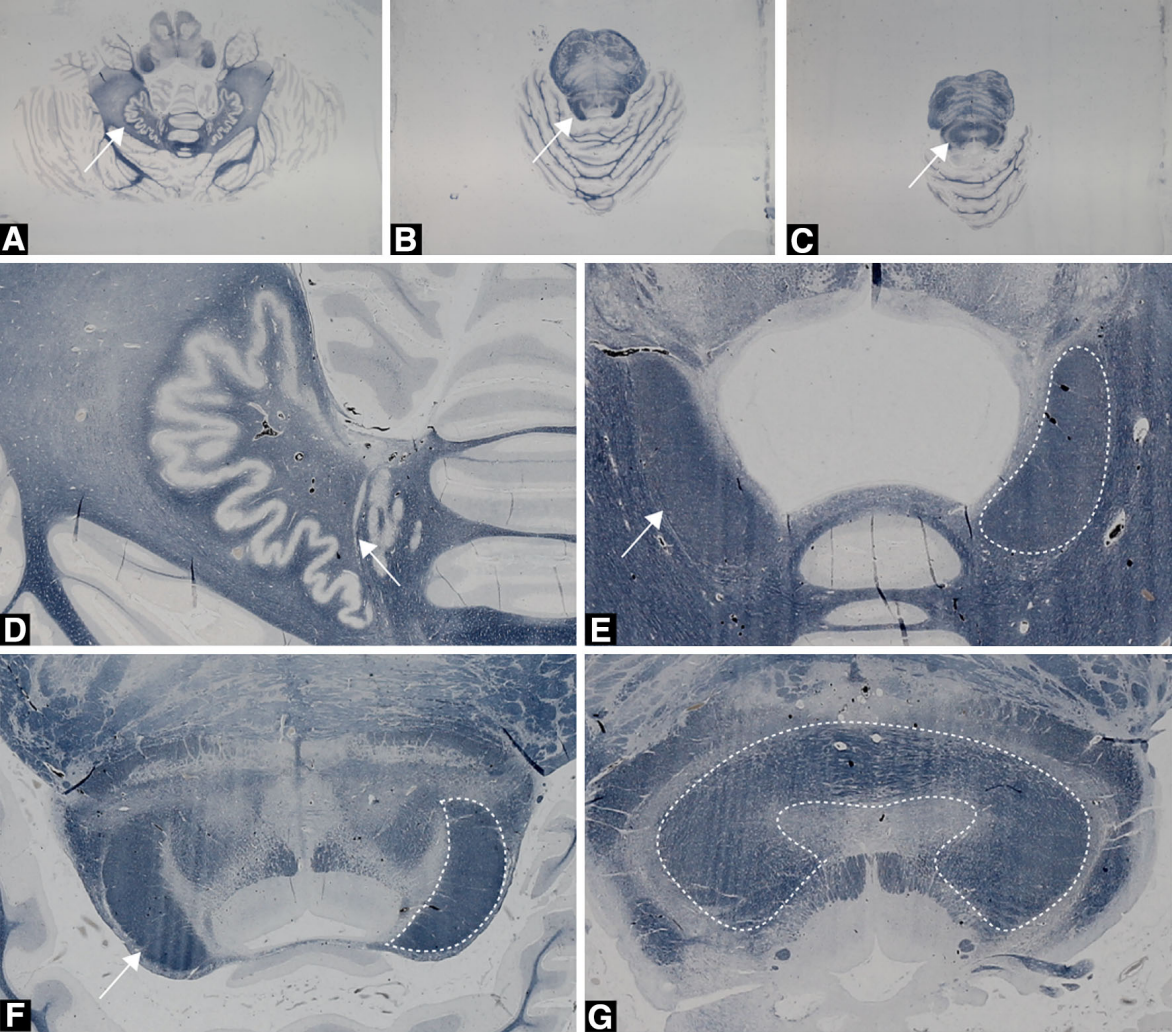
Histological axial sections stained for myelin with the modified Heidenhain–Woelke stain at different levels of the cerebellum and the pons. **a** The unstained dentate nucleus (*white arrow*) is clearly visible and characterized by its dented pattern. **b** The wedge-shaped structures represent the DRTTs (*white arrow* for left DRTT) in the superior cerebellar peduncles. **c** The decussation (*white arrow*) of left and right DRTTs at the level of the mesencephalon. **d** Close-up from **a** of the left dentate nucleus. The white matter enclosed by the dentate nucleus forms the origin of the DRTT. **e** Close-up of the DRTT within the superior cerebellar peduncles. Densely packed white matter is surrounding the DRTT at this level. Difference in texture allowed for differentiation of the DRTT with adjacent white matter indicated by the *white arrows*. The *dashed outline* marks the right DRTT in this section. **f** Close-up from **b** of the DRTT (*white arrow* and *outline*, for left and right DRTT, respectively) within the superior cerebellar peduncle, but more superior located as in (**e**). **g** Close-up from **c** of the decussation (*white outline*) of the superior cerebellar peduncles in the mesencephalon

**Fig. 4:**
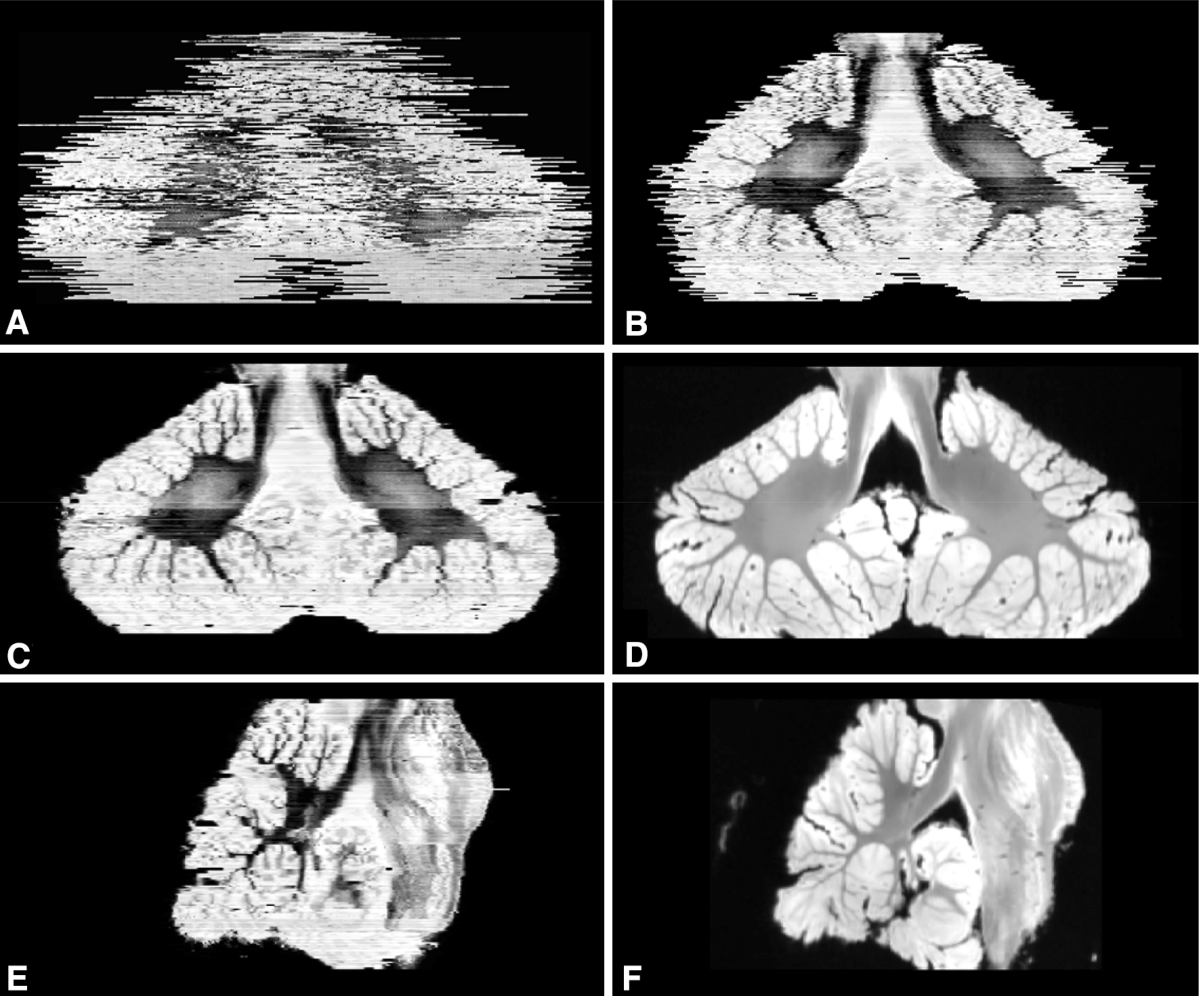
3D reconstruction of the histological sections from the cerebellum and brainstem. **a**, **b** Coronal views from before and after affine registration of the stacked slices, respectively. Following affine registration, a non-linear registration approach was applied and represented in a coronal (**c**) and sagittal view (**e**). Alignment of internal structures significantly improved after this step and was most pronounced in white matter. Corresponding MRI slices for **c** and **e** are depicted in **d** and **f**, respectively

**Fig. 5:**
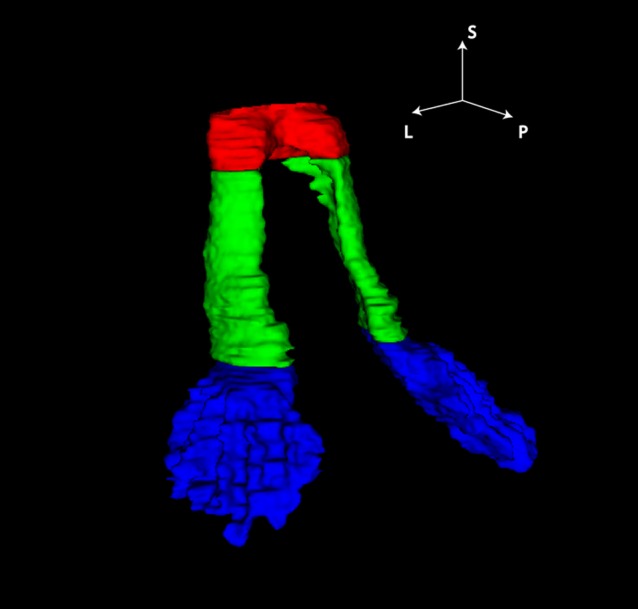
DRTT segmentation from 3D histological reconstruction. ROIs of the DRTT are labelled in: *blue* dentate nucleus; *green* superior cerebellar peduncle and *red* decussation in the mesencephalic region. Orientation labels *S* superior, *P* posterior, *L* left

The modified Heidenhain–Woelke appeared to properly stain the DRTT and provided good contrast between the DRTT and adjacent structures (Fig. 3). As expected, deformations were observed in the histological sections due to tissue processing and microtomy. These deformations were fairly severe in some cases illustrating the necessity of spatial correction for accurate 3D reconstruction. The 3D reconstruction after an affine registration scheme only produced reasonable results in terms of global alignment of the slices, but misalignment of internal structures was clearly noticeable. The diffeomorphic registration approach significantly reduced misalignment between the slices as it aims to warp corresponding structures to each other (Fig. 4). Segmentations of the DRTTs were obtained from the histological 3D reconstruction (Fig. 5).

**Fig. 6:**
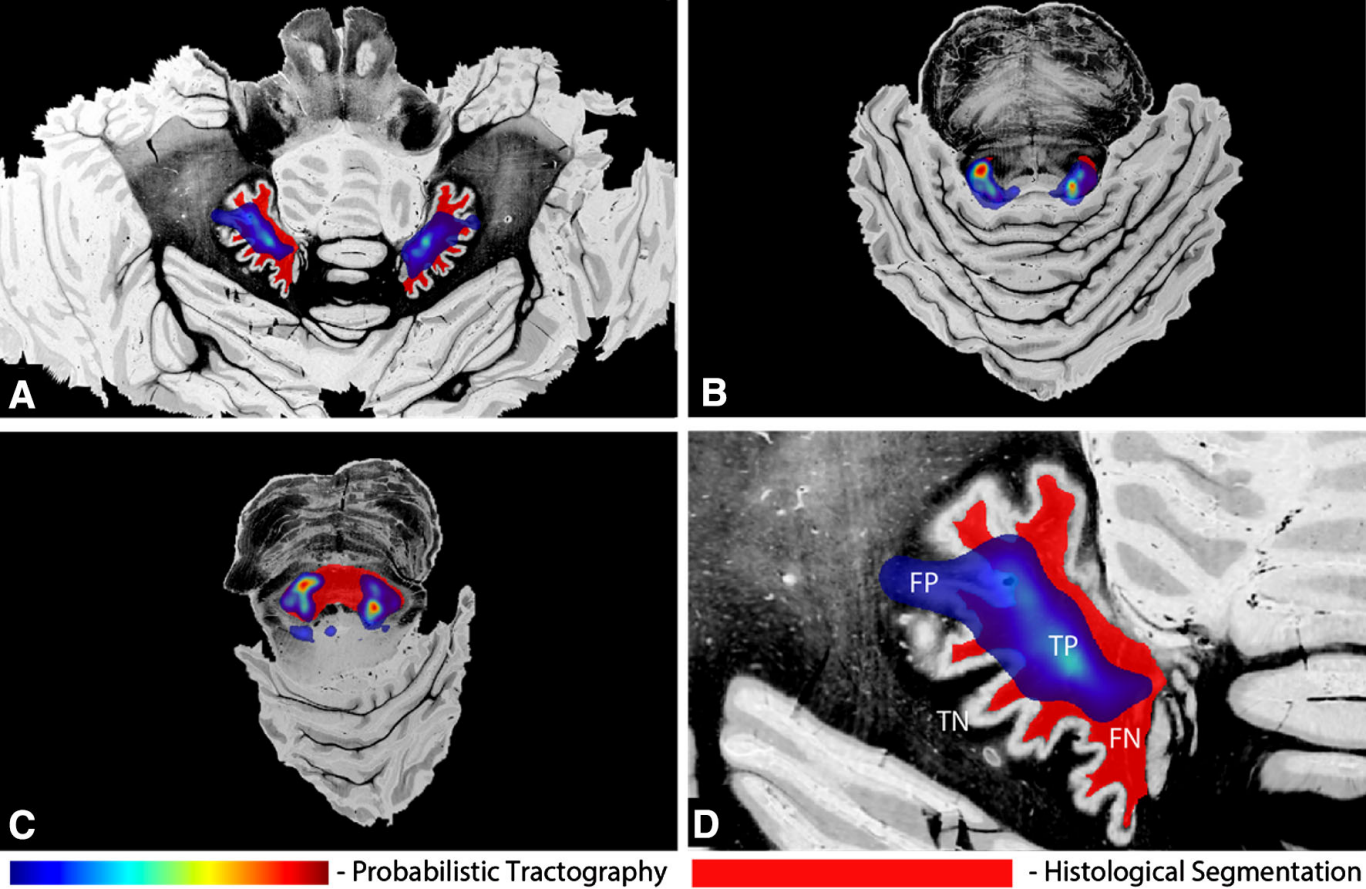
Tractography (*blue* at arbitrary threshold) compared by overlaying tractography with the histological segmentation of the tract of interest (*red*) in the axial slices of the histological volume. Regions of the dentate nucleus (**a**), superior cerebellar peduncle (**b**) and decussation in the mesencephalon (**c**) are depicted. **d** ROI of the dentate nucleus with measures for ROC analysis; *TP* true positives, *FP* false positives, *TN* true negatives, *FN* false negatives

**Fig. 7:**
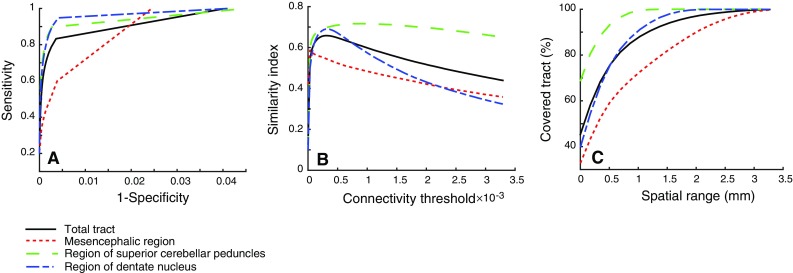
ROC curves for total tract and separate regions averaged for both *left* and *right* DRTT. **b** Similarity index as a function of the connectivity threshold. **c** Spatial extent of the FNs at optimal threshold. The optimal threshold is defined as the threshold where the similarity index is maximal

Overlaying the segmentation and binarized tractography maps thresholded at a set of connectivity values allowed calculation of the overlap characteristics (TP, FP, TN, FN: Fig. 6). ROC analysis indicates worse performance (i.e. a lower area under the ROC curve) in the decussation in the mesencephalic region as compared to the other regions (Fig. 7a). SIs were calculated as a function of threshold. An optimal threshold was defined where SI is maximal in the superior cerebellar peduncle (Fig. 7b; Table 2). This optimal threshold was 0.1 %, i.e. voxels that had at least 0.1 % of the total number of streamlines generated between the seed and target mask were included in the optimal tractography mask. The maximal SI for the superior cerebellar peduncles together was found to be 0.72.

**Table 2:** Maximum values for the similarity indices

	Total tract	Mesencephalic region	Superior cerebellar peduncle	Dentate nucleus
Left DRTT	0.65	0.62	0.68	0.68
Right DRTT	0.66	0.56	0.77	0.71
Both DRTTs	0.66	0.59	0.72	0.69

Additional analysis included the application of the optimal threshold to the tractography connectivity maps, resulting in an optimal tractography mask. Dilating this mask with a margin of 1 mm included 95 % of all DRTT fibres within the dentate nucleus and superior cerebellar peduncle (Fig. 7c).

## Discussion

For the first time, a combination of probabilistic tractography, a myelin-stained 3D tract reconstruction and spatial metrics have been demonstrated to study the anatomical accuracy of tractography in a white matter bundle: the dentatorubrothalamic tract. A good agreement between tractography and the 3D segmentation was demonstrated by the method presented here.

The modified Heidenhain–Woelke stain (Bürgel et al. 1997; Holl et al. 2011) provided excellent contrast between the DRTT and adjacent tracts. Even in regions with similar colour intensity, histological slices provided a texture contrast that allowed distinction of the DRTT from fibre bundles in close proximity (Fig. 3d). To date, MRI is unable to provide such detail.

Visual comparison of tractography results and histology results showed a fairly good agreement. An ROC analysis was conducted to quantify the results. High sensitivity in the ROC curve corresponds to a high number of voxels overlapping in the binarized tractography and histology masks relative to the total number of histology voxels. Higher thresholds on the tractography connectivity maps will result in a lower number of tractography voxels, with lower sensitivity and higher specificity. Specificity in this context represents the number of true tract-negative voxels relative to the total number of histology-negative voxels. Specificity is thus influenced by the size of the compared volumes or rather, the number of zeros in these binary masks. Therefore, another quantitative measure, the Dice similarity index (SI), was introduced to evaluate tracking results more informatively. Our results demonstrate a maximum SI of 0.72 at the level of the superior cerebellar peduncle. Less overlap was found in the decussation of the DRTTs (Fig. 7b). This can be explained by the profuse mingling of fibres from left and right superior cerebellar peduncle (Fig. 3e), prohibiting separate segmentation of the two bundles at this point. Therefore, the whole decussation was taken as reference in the analyses for both the left and the right DRTT (Fig. 6c). This assumption inevitably led to relatively many false negatives in the decussation, but it may be argued whether these were truly false negatives.

It is worth noting that the histological sections suffer from deformations when compared to their MRI reference slices. These deformations arise from various causes including histological preparations such as fixation, dehydration, embedding and microtome cutting (Dauguet 2011). Tissue deformations were inevitable and were more pronounced in the cerebellar cortex than in cerebellar white matter. A non-linear registration approach was chosen to effectively correct for these deformations. Alignment of the non-linear histological reconstruction was significantly improved when compared to the histological reconstruction after affine co-registration of the slices (Fig. 4), in particular for white matter regions. The ANTs registration toolbox allowed for this reconstruction by modelling a multivariate normalization approach. The multivariate normalization included not only the reference MR slice for each moving histological slice, but also two neighbouring histological slices to correctly align structures within the cerebellum. Such approach was previously applied in a volumetric reconstruction of the hippocampal sections (Adler et al. 2014) and deemed appropriate for the data presented here.

The results of this study implicate that postmortem reconstruction of the DRTT with the use of tractography fairly well represents its true anatomical position. Tractography may well be used as a way to visualize this tract in vivo in patients and controls and to study differences in volume or integrity of the white matter tracts. This will provide valuable information on the working mechanism of the cerebellum and the anatomical substrate and even pathophysiology of non-motor cerebellar disorders. In future, this knowledge may lead to better treatment options or even prevention of devastating syndromes such as the cerebellar mutism syndrome, for example by changing the surgical approach, trying to avoid the regions that appear most vulnerable.

When comparing groups of patients and normal controls, errors in the reconstruction of the tract (caused by region of interest selection, transformation and the reconstruction algorithm itself) will be averaged out by the group-wise approach and will not have a significant impact on the outcome (van Baarsen et al. 2015). Information on cerebellar structure–function correlations, as derived from large trials, may lead to modifications in the surgical approach of cerebellar lesions. A personalized surgical approach based on tractography in the individual patient might be a future possibility, although current evidence is insufficient to support this.

The results presented here are not easily extrapolated to a clinical situation, where diffusion protocols take 20 min rather than 32 h. In this respect, it should also be considered that postmortem DWI is different from conventional in vivo DWI. Fixed brains experience alterations in tissue properties owing to tissue degeneration after death and protein cross-linking caused by the fixative. Reduced apparent diffusion coefficient (ADC) (Sun et al. 2003) and T_1_- and T_2_ signal (D’Arceuil et al. 2007; McNab et al. 2009; Pfefferbaum et al. 2004) are noticeable MR effects due to these alterations. It is suggested that the reduction in T_2_ is at least partly driven by the presence of the fixative in tissue (Miller et al. 2012). In the present study, the specimen was soaked in phosphate-buffered saline prior to scanning to counteract the decrease in T_2_ relaxation (Shepherd et al. 2009). Soaking of the specimen might be an explanation for the intensity inhomogeneity that is observed in our data (Fig. 1). No signs of pathology were found after microscopic inspection of histological sections. The immersion time of 72 h may not have been sufficient to reach inner regions.

Although the tissue alterations are problematic for diffusion imaging, postmortem DWI certainly has some advantages compared to in vivo DWI. Lower image distortion and a higher resolution (spatial and angular) are the main benefits of postmortem DWI, because there is basically no restriction on scan time. For the DW-SSFP sequence in particular, characterized by short *T*_R_ and the use of a single diffusion-encoding gradient (McNab et al. 2009), diffusion weighting is acquired without requiring long *T*_E_. The sequence is unsuitable for in vivo DWI, because of its high sensitivity to motion, which is clearly excluded in postmortem tissue. In addition, it has been demonstrated that DW-SSFP performs significantly better than a conventional DW spin echo sequence in postmortem brains (Miller et al. 2012).

It may be argued whether the good agreement between tractography and histology as found in this study, may be generalized towards validity of tractography for the DRTT. Ideally, tractography should have been verified in more than one subject and both in vivo as well as postmortem. The latter was previously followed in animal studies, but is infeasible in human tissue due to ethical considerations.

Nevertheless, regarding the fact that a voxel-by-voxel comparison was done for both left and right DRTT, the large number of measurements was deemed adequate to reliably compare the two modalities. Whereas the individual anatomy may vary between subjects, the physical characteristics that tractography and histology are based on are probably similar among different subjects. Tractography is based on the diffusion of water molecules in white matter and myelin staining is based on the presence of lipoproteins in the neurokeratin skeleton of myelin sheaths (Bürgel et al. 1997). The overlap between tractography and histology depends on these tissue characteristics, not on the individual anatomy and will probably be of similar extent in different subjects.

Alternatively, validation of postmortem tractography could be accomplished following a step-wise approach, in which the first step would be to investigate the anatomical correspondence of postmortem tractography, as was done in this study. The second step would comprise a comparison between in vivo and postmortem tractography in non-human species followed by in vivo tractography studies in multiple subjects to address the question of inter-individual anatomical variation. In this respect, Takahashi et al. already demonstrated the ability to reconstruct the superior cerebellar peduncle in postmortem high angular resolution diffusion imaging datasets in both adults (Takahashi et al. 2013) and during developmental stages (Takahashi et al. 2014). Further, the DRTT was studied in 15 healthy subjects and was successfully reconstructed with tractography in all subjects using a 1.5 T MRI scanner (Kwon et al. 2011). Similar studies involved mapping of the DRTT in healthy subjects (Palesi et al. 2014) and patients with cerebellar lesions (Marek et al. 2015; van Baarsen et al. 2013) at 3T. Although anatomical verification is lacking in these studies, in general tractography seems to follow the known anatomy of the DRTT as described by textbooks (Nieuwenhuys et al. 2008). Indeed, a probabilistic white matter atlas of the cerebellum, based on high-quality, high-resolution data acquired from 90 subjects participating in The Human Connectome Project will address the inter-individual variability and will be published shortly (van Baarsen et al. 2015).

It should be stated that the results of this study are only valid for the DRTT and cannot be extrapolated to other white matter tracts. However, combining probabilistic tractography and three-dimensional histological tract reconstruction into an ROC approach, as demonstrated in this work, may be adopted to evaluate tractography accuracy in other white matter tracts.

## Conclusion

Tractography of the dentatorubrothalamic tract, which is the main output tract of the cerebellum, has a fairly good spatial overlap with its histological three-dimensional reconstruction. Although this may not be extrapolated to a clinical decision-making situation, it does support tractography as a reliable tool for tract localization in experimental studies.

Regarding non-motor cerebellar syndromes, reconstruction of the DRTT with tractography in patients versus controls may help in the search for their anatomical substrate.

## Electronic supplementary material

Below is the link to the electronic supplementary material.

Supplementary material 1 (EPS 18497 kb) Post mortem human brain specimen from a sided (A) and front view (B). All cerebral lobules except the insulae were removed

Supplementary material 2 (EPS 4057 kb) Tractography masks from a coronal view. (A): Seed masks in the dentate nuclei. (B): Thalamic waypoints. (C): An exclusion mask midsagittal through the specimen
